# Teaching radiology in undergraduate programmes in medical schools in francophone sub-Saharan Africa : a multicenter survey

**DOI:** 10.1186/s12909-026-09165-z

**Published:** 2026-04-09

**Authors:** Pihou Gbande, Tsatsou Alfred Koudjo Tamekloe, Mazamaesso Tchaou, Lantam Sonhaye, Lama Kegdigoma Agoda-Koussema, Komlanvi Adjenou

**Affiliations:** 1 Department of Radiology and Medical Imaging, University of Lomé, Lomé, Togo; 2 Department of Radiology and Medical Imaging, University of Kara, Kara, Togo

**Keywords:** Education, Radiology, Medical students, Francophone sub-Saharan Africa

## Abstract

**Background:**

Harmonisation of radiology education for medical students is a current global topic, particularly in Africa. The main objective of this study was to assess radiology training programmes for medical students in francophone sub-Saharan African medical schools.

**Materials and methods:**

This was a cross-sectional descriptive study conducted between May and June 2024, using a semi-structured survey. In this study, we included medical faculties training physicians in French-speaking sub-Saharan Africa, where a radiology Department member provided informed consent to participate and completed the questionnaire.

**Results:**

The study received responses from 11 faculty members representing medical schools in 9 Francophone sub-Saharan African countries. Theoretical teaching begins during the bachelor’s programme in 63.6% of faculties. A national syllabus similarity was reported in 42.9% of the cases. The average number of hours allocated to theoretical teaching was 59.7 ± 29.7 h and a median of 60 h. Medical imaging of pathological conditions was reported to be taught in all faculties (100%), followed by basic radiological semiology (72.7%). Lectures were used in all faculties (100%). Radiology internships were reported as mandatory in 90.9% of the faculties and begin during the master’s programme in 50%, with a total average duration of 6.5 ± 4.4 weeks and a median duration of 4 weeks. A written examination was reported to be mandatory in 90.9% of faculties, and internship validation was required in 81.8%.

**Conclusion:**

It appears necessary to rethink radiology education in most medical schools and to harmonise objectives and programmes in francophone sub-Saharan Africa.

**Supplementary Information:**

The online version contains supplementary material available at 10.1186/s12909-026-09165-z.

## Background

Teaching is a complex mix of pedagogy, assessment, and administration, guided by beliefs about what should be taught, when it should be taught, and how to convey information [1]. Consequently, there are numerous opinions in the literature on what constitutes good teaching [2–5]. These studies align with a quality-driven approach aimed at the continuous improvement of education.

Radiology is an essential component of medical undergraduate training, ranging from preclinical studies to clinical placements. However, medical school curricula have not been standardised, particularly with regard to teaching of radiology to medical students [6–8]. Although several frameworks have been proposed by professional societies, medical schools continue to develop their own approaches to medical education, leading to significant variations, sometimes within the same country [9, 10]. Therefore, the need to develop a formal curriculum to teach radiology and medical imaging has been established [11, 12].

In Francophone Africa, the West African Health Organisation (WAHO), a specialised institution of the Economic Community of West African States (ECOWAS), collaborates with various partners, including the African and Malagasy Council for Higher Education (CAMES), to develop and implement harmonised general medicine training curricula. The medical program has the overall objective of training professionals capable of practicing medicine in accordance with the ECOWAS regional health standards. Its structure is based on the Bachelor’s-Master’s-Doctorate (BMD) system. The BMD system is an organizational framework for higher education adopted in many countries, particularly in Europe and Africa, aimed at harmonizing academic degrees and facilitating student mobility [13].

The Francophone African Society of Radiology (SRANF), which serves as a platform for exchange between radiologists, including university professors, contributes to the promotion of radiology development in Francophone African countries and medical education through meetings and scientific training. Establishing a standardised content base for teaching radiology in medical schools is crucial. However, there are no concrete data regarding the number of hours devoted to teaching radiology or the content of these training programmes. In addition, questions remain about whether this education meets international standards in francophone sub-Saharan Africa.

The main objective of this study was to assess how radiology and medical imaging are taught to medical students in Francophone sub-Saharan Africa by estimating the theoretical and practical teaching hours allocated, examining the content of training programmes, and comparing teaching methods and assessment approaches.

## Materials and methods

### Study design and framework

This was a descriptive cross-sectional study with both quantitative and qualitative components, conducted in 9 French-speaking sub-Saharan African countries between May and June 2024.

### Study population

The target population consisted of medical faculties in French-speaking sub-Saharan African countries. Medical faculties that train general practitioners and where one radiology Department member provided informed consent and completed the questionnaire were included in the study.

### Data collection

The study was conducted using a pre-established questionnaire. The questionnaire was pre-tested, reviewed, corrected, and readjusted by two university professors to ensure clarity and comprehension. The questionnaire used in this survey is available in the supplementary files section. Responses were recorded through two methods: electronically via Google Forms and physically on printed questionnaires. Semi-structured interviews were carried out. Initially, participants completed the questionnaire, followed by discussions with some respondents to clarify their answers and fill in any missing information.

An exhaustive sampling of CAMES member countries was performed, followed by a random selection of medical faculties, with a maximum of two faculties chosen per country when applicable. The questionnaire was subsequently sent to one of the radiology lecturers, who is a member of the faculty, resulting in 18 respondents representing 18 faculties from 16 countries.

The questionnaire is divided into two main sections:

- Section 1: Focused on the respondent’s profile and the medical faculties in their country, including the number of faculties training general practitioners and the extent of curriculum similarity across faculties within the same country.
- Section 2: This section addressed teaching and assessment. Variables explored included the target audience for teaching, the theoretical and practical teaching hours, the content of the training, teaching methods, the accessible imaging modalities for students, and the assessment of theoretical and practical learning.

### Data processing and analysis

The data were processed using Excel 2016. Categorical variables were expressed as counts and proportions, while numerical variables were presented as means (SD) and medians (IQR).

### Ethical considerations

The study was conducted in accordance with the principles of the Declaration of Helsinki. Confidentiality was strictly maintained. The participants were informed that completing the questionnaire implied consent to participate in the study and to the publication of the results. This study received ethical approval from the University of Lomé Institutional Review Board (approval number: 0015/UL/CE-FSS/2024).

## Results

During this survey, 11 out of the 18 teachers responded to the questionnaire, representing 11 medical faculties, resulting in a response rate of 61.1%. These responses came from 9 out of the 16 French-speaking sub-Saharan African countries within the CAMES framework. The surveyed countries were Benin, Burkina Faso, Cameroon, the Central African Republic, Congo-Brazzaville, Côte d’Ivoire, Guinea-Conakry, Mali, and Togo.

### Theoretical teaching of radiology to medical students

Radiology teaching was reported to begin during the bachelor’s programme in seven faculties (63.6%) and during the master’s programme in four faculties (36.4%). In four faculties (36.4%), radiology is taught for only one academic year.

Seven countries have at least two medical schools, with curriculum similarity rapported in three countries (42.9%). No faculty member reported being invited to teach radiology within the curricula of other medical disciplines for medical students. The number of hours allocated to theoretical teaching in the faculties ranged from 30 to 130 h, with a mean of 59.7(29.7) hours and a median of 60 (10) hours. Medical imaging of pathologies is reported to be taught in all faculties (100%), followed by basic radiological semiology in 72.7% (*n* = 8).

Lectures are offered in all faculties (100%). It was reported that, in seven faculties (63.6%) students present oral reports. Students are involved in scientific research in four faculties (36.4%). Examples of scientific activities include oral presentations at conferences, team participation in thesis data collection, article writing, and thesis defences in radiology. Online teaching through the Moodle platform was reported in two faculties (18.2%). The projection during lectures was reported in all faculties (100%), and electronic course materials are available to students in ten faculties (90.9%). Other resources are described in the Table 1.

**Table 1:** Years, content, and teaching resources

	Counts	Percentage
Teaching Duration (Years)		
One	4	36.4
Two	3	27.2
Three	2	18.2
Four	1	9.1
Six	1	9.1
Content of Teaching		
Basic physical principles	6	54.5
Basic radiological semiology	8	72.7
Medical imaging of pathological conditions	11	100
Radiation protection	7	63.6
Teaching Resources		
Image projection during lectures	11	100
Electronic course materials	10	90.9
Image banks available to students	7	63.6
Paper-based resources	6	54.5
Audio-visual course projections	4	36.4

### Practical radiology training

Radiology internships were reported to be mandatory in 10 faculties (90.9%). Among these ten faculties, internships are limited to a single year in seven faculties (70%) and begins during the master’s program in five faculties (50%). The total duration of radiology internships during general medical training ranges from two to twelve weeks, with a mean of 6.5 (4.4) weeks and a median duration of 4 (5) weeks. It was reported that students always have access to radiography during their internship in all faculties (100%), followed by CT scans in 81.8% of cases (Table 2).

**Table 2:** Imaging modalities accessed during internships

	Counts	Percentage
Radiography	11	100
CT scans	9	81.8
Ultrasound	8	72.7
Magnetic Resonance Imaging	4	36.4
Interventional Radiology	0	0
Nuclear Medicine	0	0

### Student assessment

The assessment of students is reported to be conducted through a written exam in ten faculties (90.9%), while one faculty (9.1%) does not evaluate students. These exams are preceded by in-class tests in six faculties (54.5%). The mandatory validation of radiology internships is reported in nine faculties (81.8%), while two faculties (18.2%) do not evaluate internships. It is reported that students who have not passed their exam may retake a resit session in nine faculties (81.8%).

## Discussion

This study represents the first multicenter investigation into the teaching of radiology to medical students in francophone Africa. According to the Quality Framework of the African and Malagasy Council for Higher Education (CAMES), teaching evaluation is part of pedagogical organisation under reference A.4.8, which includes five criteria [14]. This study aims to guide universities, national committees and boards worldwide in developing and implementing improvements in medical school training.

This study demonstrates that radiology education begins during the bachelor’s programme in nearly two-thirds of the medical faculties and during the master’s programme in more than one-third of the faculties. Early exposure during medical training increases the success rates in this discipline [15, 16]. Studies have also shown that early exposure to radiology education helps students become familiar with the discipline, improves their perceptions of radiology, and increases their interest in choosing radiology as a career [17–19]. The syllabi and distribution of radiology teaching throughout the medical curriculum are reported variably in this study. Indeed, radiology education is limited to a single academic year in more than one-third of the faculties, with syllabus uniformity observed in half of the cases within the same country.This finding is consistent with the literature that indicates that radiology training, curriculum design, and pedagogy differ significantly between schools [1, 20]. Some authors have recommended that radiology teaching should achieve specific objectives based on the level of study and therefore extend over several years [21–23].

There is a significant disparity in the theoretical teaching hours across medical schools, ranging from 30 h to 130 h, with an average of 59.7 h and a median of 60 h. This result is similar to that found in a Scottish study, which reported an average of 59 h [24]. Kourdioukova et al. [25], in a comparative international study of undergraduate radiology programs in Europe, reported an average of 89 h and a median of 76 h. This demonstrates that radiology teaching is under-represented. The lack of standardisation and insufficient time allocated to radiology in the current medical curriculum is evident, considering its pivotal role in medicine. The reasons are likely numerous, including limited time within already crowded curricula, lack of access to specialized equipment, and shortage of instructors. Indeed, studies report that improving radiology education in Africa faces significant challenges, notably limited funding, faculty shortages, and insufficient availability of equipment [26–28]. Inadequate resources and infrastructure, coupled with the absence of simulation-based learning, further hinder the development of students’ practical competencies [29–31]. Overcoming these barriers requires coordinated strategies to strengthen infrastructure, enhance faculty capacity, and integrate innovative pedagogical approaches.

Regarding the content of the teachings, this study shows that radiology of pathologies is taught in all faculties, and basic radiological semiology in nearly three-quarters of the faculties. Basic semiology is taught following a review of radiological anatomy. Fundamental anatomy is still taught by anatomists and does not incorporate radiological images [32]. In some European countries, studies have shown a significant increase in the teaching of radiological anatomy in medical faculty curricula, with greater involvement of radiologists in teaching anatomy [33–35]. The combination of radiological resources with traditional anatomy teaching methodology in a unified approach would be more beneficial [34]. Radioprotection and the physical foundations of medical imaging are taught in more than half of the faculties. The teaching of the physical foundations covers all imaging modalities, including those not available in practice.This indicates that the content of radiology courses is not always fully covered according to WAHO recommendations [36]. Medical students do not receive sufficient training in radiology. Harmonizing the content of radiology education for medical students in French-speaking sub-Saharan African countries is therefore essential to ensure a uniform and high-quality training. This can be achieved by developing a common educational framework and standardized course materials, defining the minimum competencies to be acquired in radiology at each level of medical education. It will also be necessary to harmonize the number of hours and evaluation methods across faculties. Nonetheless, establishing core competencies in radiology that are aligned with employer expectations represents a crucial step toward the harmonization of curricula and the strategic planning of future training programs.

In the present study, lectures are used in all faculties, while presentations are employed in nearly two-thirds of faculties, and scientific research is incorporated in more than a third of faculties. Practical internships or observation periods in radiology are mandatory in more than four out of five faculties and are generally completed within a single year. In Egypt, Badawy et al. [37] reported 62.7% of lectures and 30.76% of self-directed learning with provided images. The literature shows that the teaching methods vary according to the level of study and the objectives to be achieved [4, 21, 38, 39]. Regardless of the method used, it should enable students to acquire knowledge that supports their clinical practice. Therefore, students should be exposed to various imaging modalities during internships. Today, artificial intelligence is gradually integrating into radiological practice. Studies have reported how it could also be incorporated in the future into various phases of student training, whether in basic education or continuing education [40–42].

The validation of radiology is mandatory through a written examination in more than three-quarters of faculties. Mandatory internship validation is also present in more than three-quarters of faculties. This evaluation includes multiple-choice questions, short-answer questions, and clinical cases. Both methods are summative assessments. A combination of summative and formative assessments, which are complementary, is essential, as they are known to facilitate student learning and understanding while helping them identify areas of weakness [43, 44]. According to Jouquan [45], in medical education, learning assessment shape the nature and quality of student learning. Students fundamentally and strategically adapt to what is expected of them; consequently, the likelihood of significantly influencing their learning is higher. Thus, assessment should be considered central to the teaching-learning dynamic. Assessment also strongly influences student motivation by providing information that improves their sense of competence to complete learning tasks.

### Study limitations

The main limitation of this study is the small sample size. Additionally, the study did not take into account the curricula or the students’ clerkship booklets. This study did not include a detailed account of students’ experiences during theoretical courses and the radiology internship, which constitutes a limitation.

## Conclusions

This survey of radiology teaching for medical students in Francophone sub-Saharan Africa highlights heterogeneity in the time allocated to teaching, the content covered, and the methods of teaching and assessment. It appears necessary to rethink radiology education in most medical schools and to harmonise objectives and programs across Francophone Sub-Saharan Africa. This will help overcome the fragmentation of medical training in general, and radiology in particular, thus facilitating the mobility of students and trained physicians between different countries. Harmonizing the content of radiology education for medical students will be essential to ensure a uniform and high-quality training, enabling future physicians to meet international standards while adapting to local specificities.

## Supplementary Information

Supplementary Material 1.

## Data Availability

The datasets used and/or analyzed during the current study are available from the corresponding author on reasonable request.

## Abbreviations

CAMES: African and Malagasy Council for Higher Education
CT: Computed tomography
ECOWAS: Economic Community of West African States
SRANF: Francophone African Society of Radiology
WAHO: West African Health Organisation
