# Supplementary material for: Teaching radiology in undergraduate programmes in medical schools in francophone sub-Saharan Africa : a multicenter survey
Source: BMC Med Educ. 2026 Apr 9;26:825. doi: 10.1186/s12909-026-09165-z (PMC13217686; doi:10.1186/s12909-026-09165-z)
Supplement: Supplementary file 1 — Supplementary Material 1. [file 12909_2026_9165_MOESM1_ESM.pdf]

SURVEY FORM

Form number................

Respondent profile

Title (Rank):

Country:

Email:

How many faculties train general practitioners in your country? List them:

In which faculty(ies) do you teach radiology?

For which faculty(ies) are you providing this information?

Are there faculties with a similar teaching curriculum?

☐ YES ☐ NO

If yes, list them:

Radiology teaching and assessment

Target Audience: At which level(s) of study is radiology taught? List them:

Do you have a syllabus? ☐ YES (if yes, please send it via email or WhatsApp) ☐ NO

How many hours are allocated to theoretical teaching each year?

Are you invited by other faculty members to teach radiology as part of their syllabus? ☐ YES ☐ NO

If so, list them:

Does the teaching include the following modules? (Check the answers)

☐ Physical basics

☐ Basic semiotics

☐ Medical imaging of pathologies

☐ Radiation protection

Do you provide in-person lectures? ☐ YES ☐ NO

Do you offer online courses? ☐ YES ☐ NO

If yes, through which platform(s)?

Radiology internship

Is an internship in a radiology department mandatory? ☐ YES ☐ NO

If so, at which level(s) of study?

What is the duration of the radiology internship per year?

What imaging modalities are available to students during their internship?

☐ X-ray ☐ Ultrasound ☐ CT Scan ☐ MRI ☐ Nuclear Medicine ☐ Interventional Radiology

Note: Your agreement to complete this form signifies your consent to participate in the survey.

Thank you for your cooperation.
